# CD4^+^ T Helper Cell Plasticity in Infection, Inflammation, and Autoimmunity

**DOI:** 10.1155/2017/7083153

**Published:** 2017-01-29

**Authors:** Samuel Huber, Nicola Gagliani, William O'Connor, Jens Geginat, Flavio Caprioli

**Affiliations:** ^1^Department of Medicine, University Medical Center Hamburg-Eppendorf, 20246 Hamburg, Germany; ^2^Department of General, Visceral and Thoracic Surgery, University Medical Center Hamburg-Eppendorf, 20246 Hamburg, Germany; ^3^Department of Immunology and Microbial Disease, Albany Medical Center, Albany, NY, USA; ^4^Istituto Nazionale di Genetica Molecolare Romeo ed Enrica Invernizzi, Milan, Italy; ^5^Gastroenterology and Endoscopy Unit, Fondazione IRCCS Ca' Granda Ospedale Maggiore Policlinico, Milan, Italy

CD4 T helper (T_H_) cells orchestrate the immune response and play a pivotal role in infection, inflammation, and autoimmunity. CD4 T_H_ cells can be subdivided into different subsets, which are defined based on a specific network of transcriptional regulators and unique cytokine profiles. This model despite its limitation has proven to be useful to understand the complexity of the immune system and its relationship to different types of immune mediated inflammatory diseases. Interestingly recent findings indicate that some T_H_ cell subsets have a certain degree of plasticity. They can share characteristics typical of other types of T_H_ cells and potentially lose their original features to convert into another T_H_ cell subset. This has been shown for all known T_H_ cell subset but best studied for T_H_17 cells [1, 2]. Thus T_H_17 cells have the capacity to acquire a T_H_1 phenotype under chronic inflammation [3, 4] but can also convert to regulatory T cells [5–9] and participate in the resolution of the immune response [5, 7–9].

These basic aspects of T_H_ cell lineages and plasticity are discussed by J. E. Belizário et al. who focused on thymic and postthymic regulation of naïve CD4^+^ T cell lineage fates in humans and mouse models. Furthermore M. L. Diller et al. described the link between T_H_17 and regulatory T cells highlighting the mechanisms driving T_H_17 cells plasticity and discussed the biologic consequences of their unique relationship.

T helper cell plasticity seems to play a key role in amplitude of diseases. Accordingly L. Barbarash et al. analyzed T cell response in patients with implanted biological and mechanical prosthetic heart valves. Their findings suggest that altered composition of T cell subsets correlates with the development of xenograft rejection. Furthermore A. Ni et al. studied T_H_17 cell response following motor nerve injury in mice. They found that motor nerve injury exacerbates T_H_17 cell responses, which may contribute to the development of amyotrophic lateral sclerosis. J. Ruhnau et al. reported reduced numbers and impaired function of regulatory T cells in peripheral blood of ischemic stroke patients. C. F. Krebs and O. M. Steinmetz review the role of CD4^+^ T cell fate in glomerulonephritis. Interestingly, T_H_17 cells seem to have a relatively stable phenotype and regulatory T cells show heterogeneity rather than plasticity during glomerulonephritis. These findings suggest that the environment plays a key role during T helper cell plasticity.

In conclusion, we hypothesize that the study of T_H_ cell plasticity could pave the way for future therapies aiming to steer an immune response towards the desired outcome. However, it is unclear at which stage of maturation T_H_ cells will lose their potential plasticity and if T cell plasticity plays an essential role during physiological immune responses or whether it is merely a tolerable “mistake” which does not provide any physiological advantage. If this latter point would turn out to be true, this will not exclude the possibility of reprogramming the immune system but this reprogramming will probably lead to more side effects.

Nevertheless, it is now obvious that we have to enlarge the original frame of the monolithic model of T helper cell subsets in order to fully comprehend the biology of CD4 T cells. Establishing a simplified model, which integrates the original knowledge and the new findings regarding plasticity, will help to predict T helper cell behavior and it will be essential to overcome the current boundaries limiting the potential clinical applications of this knowledge.

*Samuel Huber* *Nicola Gagliani* *William O'Connor Jr.* *Jens Geginat* *Flavio Caprioli*
